# Cerebellar ependymoma with overlapping features of clear-cell and tanycytic variants mimicking hemangioblastoma: a case report and literature review

**DOI:** 10.1186/s13000-017-0619-2

**Published:** 2017-03-20

**Authors:** Xiu-Peng Zhang, Yang Liu, Di Zhang, Qin Zheng, Chen Wang, Liang Wang, Qing-Chang Li, Xue-Shan Qiu, En-Hua Wang

**Affiliations:** 1 Department of Pathology, the First Affiliated Hospital and College of Basic Medical Sciences, China Medical University, Shenyang, 110001 China; 2 Institute of Pathology and Pathophysiology, China Medical University, Shenyang, 110001 China

**Keywords:** Clear-cell ependymoma, Tanycytic ependymoma, Hemangioblastoma, Cerebellum, Epithelial membrane antigen, Case report

## Abstract

**Background:**

Imaging and histology of clear-cell ependymoma and cerebellum-based hemangioblastoma are similar; distinguishing between them is a diagnostic challenge.

**Case presentation:**

A 62-year-old Chinese woman presented with an intermittent headache of 8 years’ duration. Computed tomography and magnetic resonance imaging revealed a mass in the cerebellum. Neurological imaging suggested hemangioblastoma (HB). Histologically, the tumor included cellular and paucicellular areas, in which cells were arranged in nests or diffusely distributed; and a highly vascular area, in which tumor cells were arranged in clusters and separated by capillaries. At low magnification, the tumor mimicked cellular HB, but at high magnification, tumor cells showed clear cytoplasm instead of the vacuolated cytoplasm typically observed in HB. Moreover, spindly, bipolar elements resembling tanycytes were observed within the nest structures. Although these features indicated the possibility of ependymoma, neither true ependymal rosettes nor an ependymal-lined profile was observed. The tumor was characterized by prominent vascularity, but glomeruloid formation was absent. We saw pleomorphism in foci of some tumor giant cells, but pathologic mitosis and palisaded necrosis were absent. Most tumor cells were positive for glial fibrillary acidic protein and S100. Epithelial membrane antigen was expressed with a paranuclear dot-like or a ring-like pattern. The Ki-67 index was approximately 2%. Considering the patient’s symptom, neurological imaging, and pathological findings, she was diagnosed as cerebellar ependymoma (WHO grade II).

**Conclusions:**

Here, we report a case of ependymoma with overlapping clear-cell and tanycytic features, and review the literature to evaluate its real incidence. Pathologists should consider this rare diagnosis when confronted with a similar presentation.

## Background

Ependymoma is a slow-growing tumor that accounts for 2–9% of all neuroepithelial tumors and 6–12% of all intracranial tumors in children [1–3]. Ependymoma can develop in all age groups with no sex predilection, but its incidence is correlated with histological type and location. Whereas infratentorial ependymomas are more frequent in children, spinal ependymomas typically develop in adults [1, 2]. Within the ventricular system and spinal canal, the 4th ventricle and spinal cord are the most common sites for this tumor, followed by the lateral ventricles and the third ventricle [1]. Rare extraneural ependymomas have reportedly originated in the ovaries, broad ligaments, peri-adnexal pelvic tissues, soft tissues, mediastinum, lung, liver and the sacrococcygeal area, although female genital tract and mediastinal cases may well represent monodermal teratomas [1, 4]. According to 2016 World Health Organization (WHO) Central Nervous System (CNS) classification, ependymomas include three histopathological variants: papillary ependymoma (PE), clear-cell ependymoma (CCE), and tanycytic ependymoma (TE) [5]. Recently, one genetically defined ependymoma subtype has been added to the 2016 CNS WHO as a novel entity: Ependymoma, RELA fusion-positive [5]. which accounts for most supratentorial tumors in children [6, 7]. Occasionally, however, ependymomas present with increased cellularity and mitotic activity, often associated with microvascular proliferation and pseudo-palisading necrosis. These tumors are designated as anaplastic ependymoma (WHO grade III), which is associated with aggressive behavior and unfavorable prognosis [1, 4]. Of the three variants, clear-cell and tanycytic ependymomas are uncommon. In contrast to the other variants of ependymomas, CCEs are associated with aggressive behavior and early recurrence, even with gross total resection, and need more active management [8, 9]. Here, we present a rare case of cerebellar ependymoma with overlapping features of clear-cell and tanycytic variants in an adult female, which mimicked hemangioblastoma (HB) on histology, thus necessitating immunohistochemistry (IHC) testing for definitive diagnosis.

## Case presentation

### Clinical history

**Fig. 1:**
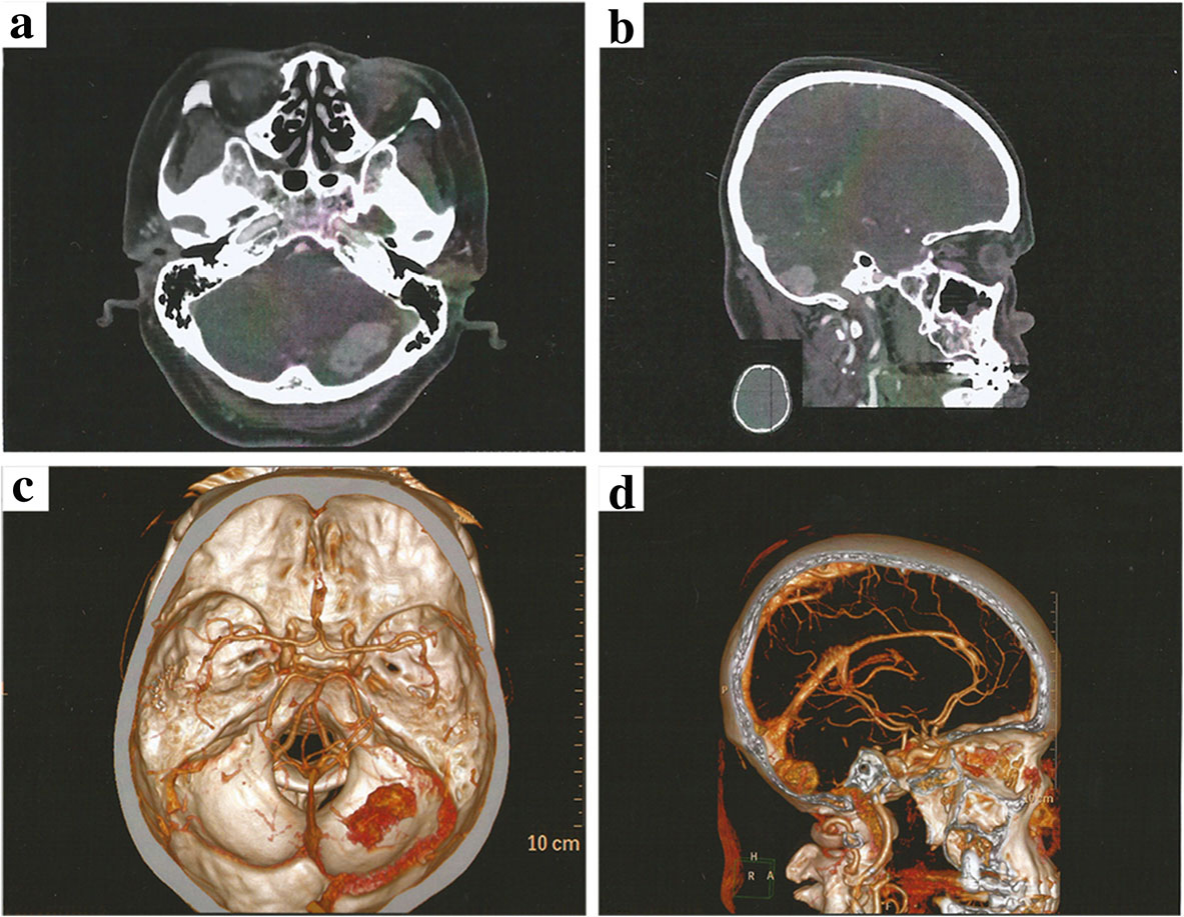
Imaging examination results. Contrast-enhanced CT reveals an oval mass with heterogeneous enhancement in the right cerebellum. (**a**: coronal view; **b**: sagittal view). CTA showed tumor with abundant blood supply (**c**: coronal view; **d**: sagittal view)

A 62-year-old Chinese woman was admitted to our hospital with a history of 8 years’ intermittent headache, which had been aggravating over the previous week. Her family history was irrelevant to the present illness. General and elemental neurological exams were normal. Contrast-enhanced computed tomography (CT; 230 HU; Fig. 1a, b) showed an oval mass (3.1 × 1.6 cm) with heterogeneous enhancement in the right cerebellum. Computed tomographic angiography (CTA) showed a highly vascular tumor; no obvious change was found on bilateral basilar arteries, vertebral arteries or posterior cerebral arteries (Fig.1c, d). Neurological imaging suggested HB. A suboccipital craniotomy of the posterior fossa was performed for tumor resection. The mass was demarcated from the surrounding cerebellum tissue with focal slight adhesion. A tumor 3.2 × 2 × 1.7 cm in size was completely resected. The patient is alive with no sign of tumor recurrence or metastasis after 9 months of follow-up.

### Materials and methods

The whole mass was completely sampled and fixed in 10% formalin, and then embedded in paraffin. A series 4-μm-thick sections was cut from each paraffin-embedded block; the top and the bottom sections were stained with hematoxylin/eosin to ensure the existence of tumor tissue, and the others in the middle were used for further IHC analyses. Commercially available prediluted monoclonal antibodies directed against epithelial membrane antigen (EMA), glial fibrillary acidic protein (GFAP), oligodendrocyte lineage transcription factor 2 (olig2), NeuN, synaptophysin, isocitrate dehydrogenase-1 (IDH1), CD56, S100, vimentin, CD34, p53, inhibin-α, D2-40, and Ki-67 were purchased from Mai Xin Inc., Fuzhou, China. IHC staining was performed using the streptavidin-peroxidase system (Ultrasensitive; Mai Xin Inc., Fuzhou, China) according to the manufacturer’s instructions. The primary antibody was replaced with phosphate-buffered saline as for negative controls.

### Chromosome 1p and 19q co-deletion analysis

We performed fluorescent in situ hybridization (FISH) to check for deletions of chromosomes 1p and 19q. Dual-color-probe hybridization was performed with Vysis 1p36/1q25 and 19q13/19p13 FISH Probe Kit (Abbott Molecular, USA) according to manufacturer’s instructions. At least 100 non-overlapping nuclei were counted; samples were considered to be 1p- or 19q-deleted when >30% of counted nuclei presented one target (red) signal and two reference (green) signals.

### Microscopic features

**Fig. 2:**
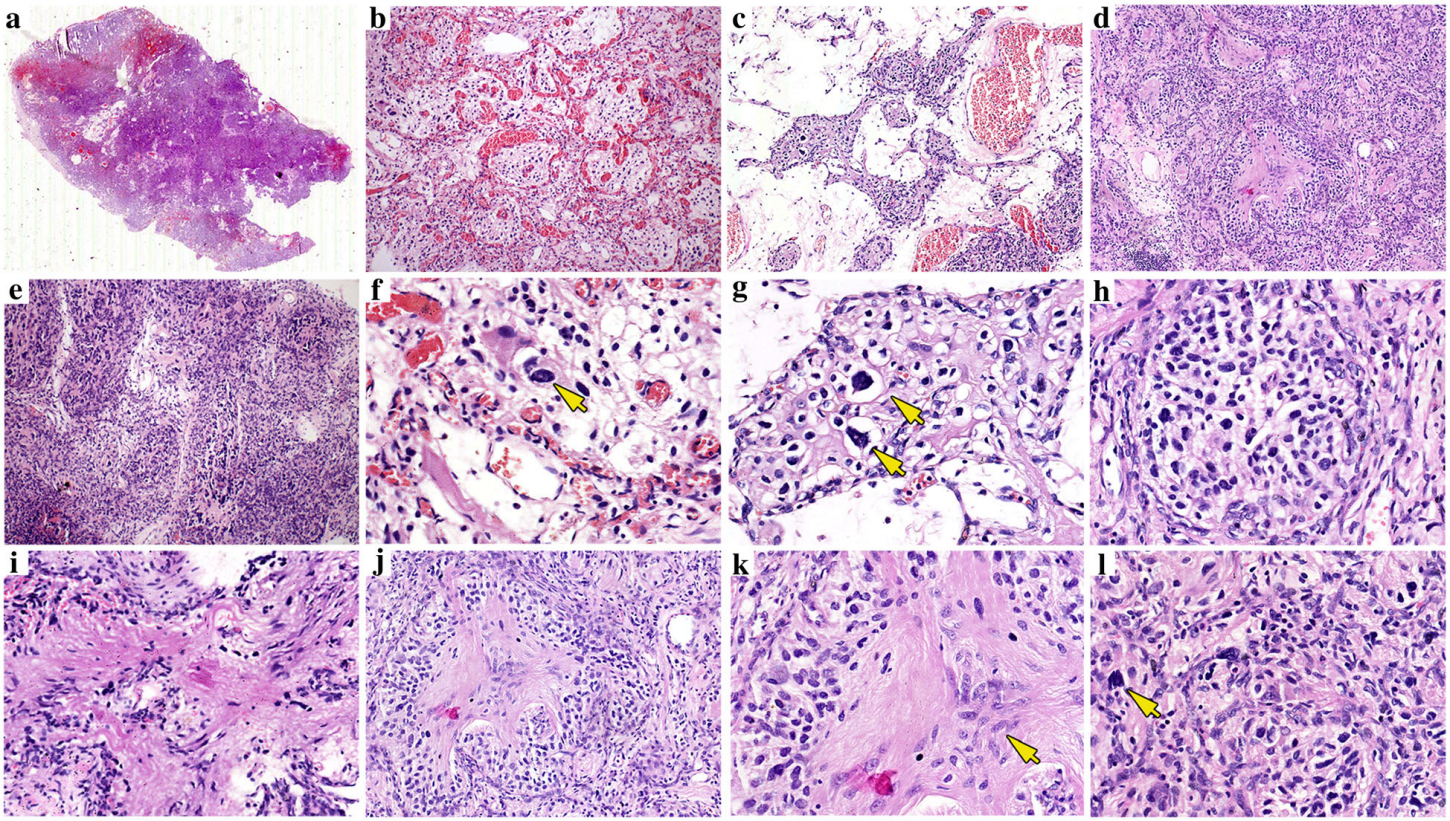
Histological features of the current case. **a**: The low magnification of the whole slide showed the tumor with a relatively clear boundary. **b**-**e**: The tumor composed of highly vascular area, cellular area and paucicellular area. **f**-**h**: In all three regions, tumor cells with clear cytoplasm could be observed. The arrows indicated the giant tumors. **i**: Foci of spindly and bipolar cells resemble tanycytes were observed within the tumor. **j**: Spindly, bipolar elements may exist in the midst of the cellular nest surrounding by the clear cells. **k**: Nuclei of these cells in the midst exhibited the salt and pepper speckling. The arrows indicated the giant tumors indicated the area of tanycytic variant. **l**: Foci of giant tumor cells with marked pleomorphism. The arrows indicated the giant tumors

Histologically, the tumor had a relative clear boundary (Fig. 2a), and composed of highly vascular, cellular, and paucicellular areas (Fig. 2b–e). In the highly vascular area, the tumor was characterized by prominent vascularity, with an abundant vascular meshwork, and clusters of clear cells that mimicked reticular HB (Fig. 1b); whereas tumor cells in the paucicellular (Fig. 2c) and cellular areas (Fig. 2d) were arranged in nests or diffusely distributed, which mimicked the cellular variant of HB. The tumor cells were characterized by clear cytoplasm (Fig. 2f–h), but the typical fat-laden vacuolated stromal cells in HB were absent. Spindly bipolar cells resembling tanycytes were also observed within the tumor (Fig. 2i), some of which were seen in the centers of nests surrounding by clear cells. This unique histological structure was detected in ~ 50% of the tumor area (Fig. 2d, j, k). Nuclei of these cells exhibited salt-and-pepper speckling with elongated cytoplasmic processes (Fig. 2k). Although these features suggested ependymoma, neither true ependymal rosettes nor ependymal-lined profile were observed. No calcification was found. The tumor was characterized by the prominent vascularity, but glomeruloid formation was absent. Despite the marked focal pleomorphism (Fig. 2l), pseudo-palisading necrosis and atypical mitoses were not present.

### Immunohistochemistry

**Fig. 3:**
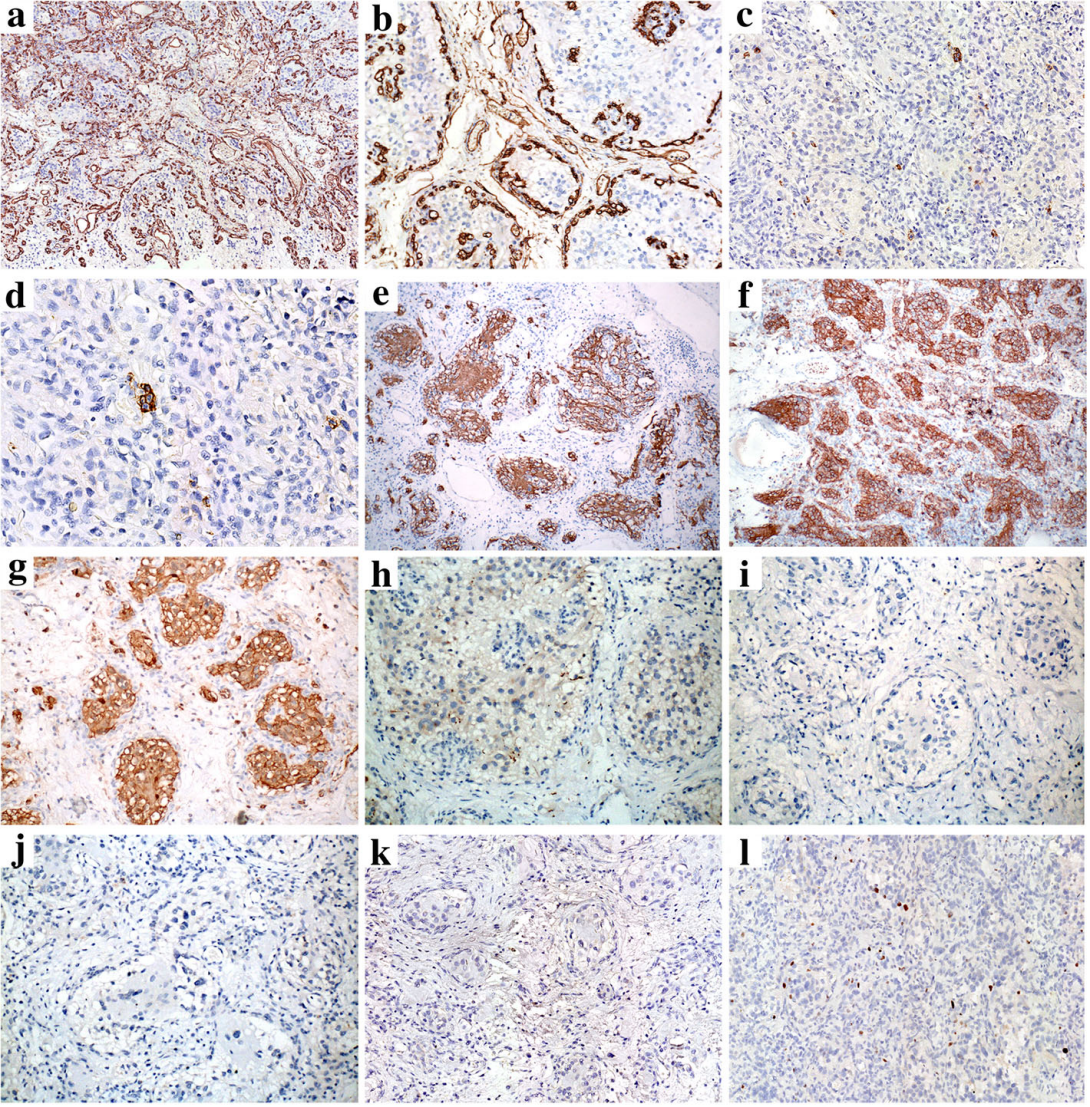
Immunohistochemical staining. **a**-**b**: CD34 underlined the rich and delicate vascular channels, while the tumor cells were negative for CD34. **c**-**d**: The tumor cells expressed EMA in a paranuclear dot-like or a ring-like pattern. **e**-**k**: The tumor cells were diffusely positive for GFAP, CD56 and S100, but negative for synaptophysin, olig2, inhibin-α and NeuN. **l**: The Ki-67 labeling index was approximately 2%

CD34 outlined the rich and delicate vascular channels (Fig. 3a, b). Epithelial membrane antigen was expressed in paranuclear dot-like or a ring-like patterns (Fig. 3c, d). The tumor cells were diffusely positive for GFAP (Fig. 3e), CD56 (Fig. 3f), S100 (Fig. 3g), and vimentin, but negative for CD34 (Fig. 3b), synaptophysin (Fig. 3h), olig2 (Fig. 3i), inhibin-α (Fig. 3j), NeuN (Fig. 3k), D2-40, P53 and IDH1. The Ki-67 labeling index was approximately 2%, even in the cellular area with prominent cell pleomorphism (Fig. 3l).

### Chromosome 1p and 19q co-deletion analysis

**Fig. 4:**
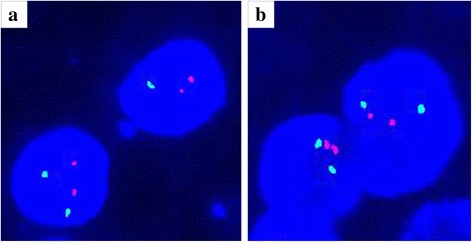
Chromosome 1p and 19q co-deletion analysis. Neither 1p (**a**) nor 19q (**b**) deletion was detected in the current case

Neither 1p nor 19q deletion was detected in the current case (Fig. 4a, b).

## Discussion

According to the 2016 CNS WHO classification, ependymomas include three histopathological variants: PE, CCE, and TE. Rare ependymoma variants include ependymoma with lipomatous differentiation, giant-cell ependymoma, ependymoma with extensive tumor cell vacuolation, melanotic ependymoma, signet-ring cell ependymoma, ovarian ependymoma, ependymoma with neuropil-like islands (ENI), and ganglioglioma with a tanycytic glial component [1]. To avoid misdiagnosis, comprehending the histological features of each variant of ependymoma is important.

In the current case, the tumor was characterized by the overlapping features of clear-cell and tanycytic variants of ependymoma. Because of the rarity of this histological feature, we reviewed literature for previously reported cerebellar clear-cell [9–14] and intracranial tanycytic [15–24] variant cases (Tables 1 and 2). However, the unique feature in this case—spindly bipolar cells surrounded by clear cells, forming cellular nests—was never described in any of these English-language studies. This novel histological pattern indicates that these two subtypes of ependymoma may coexist in the same tumor.

**Table 1:** Summary of previously reported cerebellar clear-cell ependymoma in the literature

Author	Year	No. of cases	Age/sex	Follow-up (months)	Outcome	Recurrence
Kawano *et al.* [10]	1989	2	26/M	144	Dead	Yes
			55/F	48	NA	NA
Kakita *et al.* [11]	1995	1	64/F	NA	NA	NA
Kawano *et al.* [12]	1999	6	50/M	0	Dead	NA
			64/M	60	Dead	NA
			29/M	120	NA	Yes
			45/M	120	NA	No
			43/M	132	NA	No
			23/M	120	NA	No
Hayashi *et al.* [13]	2005	1	67/M	NA	SD	No
Kim *et al.* [14]	2007	1	3/F	10	NED	No
Nagamatsu *et al.* [9]	2009	1	57/M	40	NA	No

*NA* not available, *NED* no evidence of disease, *SD* stable disease

**Table 2:** Summary of previously reported intracranial tanycytic ependymoma in the literature

Author	Year	No. of cases	Age/sex	Location	Follow-up (months)	Outcome	Recurrence
Friede *et al.* [24]	1978	4	3.5/M	the fourth ventricle	NA	NA	NA
			10/M	the fourth ventricle	NA	NA	NA
			34/M	the fourth ventricle	NA	NA	NA
			75/F	foramen of Monro	NA	NA	NA
Daneyemez *et al.* [15]	1999	1	42/M	right lateral ventricle	36	NED	No
Hayashi *et al.* [16]	2000	1	51/M	right frontal lobe	14	NED	No
Richards *et al.* [17]	2004	1	17/M	subcortical white matter of the left frontal lobe	13	NED	No
Ragel *et al.* [18]	2005	1	55/F	the third ventricle	3	SD	No
Zhang *et al.* [19]	2008	1	38/M	right lateral ventricle	8	SD	No
Du *et al.* [23]	2009	1	36/Male	Lateral ventricle	NA	NA	NA
Arvanitis *et al.* [22]	2013	1	40/M	lateral ventricle-anterior horn	NA	NA	NA
Agarwal *et al.* [20]	2014	1	44/F	the third ventricle	12	SD	No
Kambe *et al.* [21]	2014	1	2/M	right parietal lobe	NA	NA	No

*NA* not available, *NED* no evidence of disease, *SD* stable disease

Because of our case’s histological similarity to CCE, we first reviewed literature on this ependymoma variant. CCE is a rare brain tumor, accounting for 0.3% of all gliomas [9]. To date (and to our knowledge), only 27 reports in the English-language literature describe 69 cases [8, 9, 12, 25–30]. CCE has a predilection for the supratentorial region compared with classical ependymoma subtypes. Approximately 12 cases reportedly developed in the cerebellum (Table 1) [9–14]. Given the similarities in neuroimaging and histology, differential diagnosis between CCE and HB is clinically challenging. Moreover, CCE often lacks ependymomatous features such as ependymal rosettes, perivascular rosettes, or ependymal canals, which makes diagnosis more complicated. In a retrospective study, eight cases that were previously interpreted as hemangioblastomas were actually CCEs [12]. In our case, the region with abundant capillary meshwork resembled a reticular HB, whereas the cellular area with clusters of clear cells could be interpreted as a cellular HB counterpart. The immunophenotype (S-100+/CD56+/NSE+) would also have supported the diagnosis of HB, if the typical paranuclear dot-like or a ring-like pattern of EMA expression had been missed without carefully observation. Cellular HB may express GFAP, but intensity is weaker and less extensive than in ependymoma. Notably, the current case showed neither the typical fat-laden vacuolation nor inhibin-α positivity of HB. Vacuolated cytoplasm may be found in CCE, but is usually limited to focal area. Therefore, the clear vacuolated cytoplasm was a diagnostic clue for the HB. The IHC panel was also helpful for the definite diagnosis, especially as paranuclear dot-like and ring-like patterns for EMA could be observed.

Compared with the other ependymomas (WHO grade II), CCE usually present with aggressive behavior and bad prognosis, with a recurrence rate of 60% [9, 31]. In addition, this variant is always accompanied with high Ki-67 index and p53 overexpression [32]. However, our patient is alive with no tumor recurrence or metastasis after 9 months of follow-up. Whether the tumor with overlapping features of both CCE and TE has a relatively favorable biological behavior compared with the typical CCE remains to be determined in the long-term follow-up.

We next reviewed the literature om TE. TEs are typically encountered at spinal levels. Intracranial TE is extremely rare; only 13 cases are currently reported (Table 2) [15–24]. Primary cerebellar TE has not been reported in English-language literature. TE usually presents with a well-demarcated mass in neurological imaging. The typical histological feature for TE is predominantly fascicular architecture with variable width and cell density. Typified by elongated spindly bipolar cells possessing thin eosinophilic fibrillary processes, it has been speculated that this variant of ependymoma resembles the primitive radial glia-like tanycytes and therefore termed as TE. In contrast to the classic ependymoma, ependymal rosettes are usually absent or only ill-defined pseudorosettes formed in TE, while the nuclei exhibit the typical feature of salt-and-pepper speckling of other variants of ependymomas. Mitoses and necrosis are hardly observed in TE. Occasionally, hyalined vessel walls and calcospherites may be observed which presents the degenerative changes.

Based on the focal nuclear pleomorphism, giant-cell ependymoma, anaplastic ependymoma or giant-cell glioblastoma should be considered into the differential diagnosis. Although the nuclear pleomorphism was obvious in focal area, neither significant mitotic activity nor pseudo-palisading necrosis were seen in the present case. In addition, the Ki-67 labeling index was low and microvascular proliferation was absent in this case. Therefore, only the nuclear pleomorphism is insufficient to render a diagnosis of anaplastic ependymoma. Similarly, the histological features also do not support the diagnosis of giant-cell glioblastoma. In addition, giant cells in current case were limited to focal areas and did not constitute the major component for rendering a diagnosis of giant-cell ependymoma.

In this case, the spindly bipolar cells that formed nests amid clear cells prompted us to consider another variant of ependymoma: ENI. The histological feature in current case was similar to ENI, both of them formed numerous islands composed of a fibrillary central core which was surrounded by small, round to oval tumor cells. The key point to distinguish between them was the histological origin of the fibrillary central core. As termed as ENI, the fibrillary central core was composed of neuropil-like matrix and should be labeled by synaptophysin and NeuN. Moreover, the small, round surrounding cells were actually neurocytic cells and therefore should also be labeled by synaptophysin and NeuN. Only sporadic reports described this seldom variant with unfavorable prognosis [33]. Unlike ENI, specimen tumor cells in this case were positive for GFAP and S100, but not synaptophysin and NeuN, which did not support the diagnosis of ENI.

Finally, oligodendroglioma should be included in the different diagnosis because of the numerous clear tumor cells. In the current case, the immunophenotype (negative for olig2, and EMA expressed in paranuclear dot-like or a ring-like patterns), together with the negative results for chromosome 1p and 19q deletion analysis, ruled out the diagnosis of oligodendroglioma.

## Conclusion

This description and review of features in this case report of cerebellar ependymoma with overlapping clear-cell and tanycytic features are intended to make pathologists aware of this rare histological feature, and help to avoid misdiagnosing this CNS tumor (WHO grade II) as HB, which is a biologically benign tumor.

## Abbreviations

CCE: Clear-cell ependymoma
CNS: Central Nervous System
CT: Computed tomography
CTA: Computed tomographic angiography
EMA: Epithelial membrane antigen
ENI: Ependymoma with neuropil-like islands
FISH: Fluorescent in situ hybridization
GFAP: Glial fibrillary acidic protein
HB: Hemangioblastoma
IDH1: Isocitrate dehydrogenase-1
IHC: Immunohistochemistry
olig2: Oligodendrocyte lineage transcription factor 2
PE: Papillary ependymoma
TE: Tanycytic ependymoma
WHO: World Health Organization.
